# Follicular Helper T Cells are Essential for the Elimination of *Plasmodium* Infection

**DOI:** 10.1016/j.ebiom.2017.08.030

**Published:** 2017-09-04

**Authors:** Damián Pérez-Mazliah, Minh Phuong Nguyen, Caroline Hosking, Sarah McLaughlin, Matthew D. Lewis, Irene Tumwine, Prisca Levy, Jean Langhorne

**Affiliations:** The Francis Crick Institute, London, UK

**Keywords:** Follicular helper T (Tfh) cells, *Plasmodium*, Vector transmission, Signaling Lymphocyte Activation Molecule (SLAM)-Associated Protein (SAP), GC B cells

## Abstract

CD4^+^ follicular helper T (Tfh) cells have been shown to be critical for the activation of germinal center (GC) B-cell responses. Similar to other infections, *Plasmodium* infection activates both GC as well as non-GC B cell responses. Here, we sought to explore whether Tfh cells and GC B cells are required to eliminate a *Plasmodium* infection. A CD4 T cell-targeted deletion of the gene that encodes *Bcl6*, the master transcription factor for the Tfh program, resulted in complete disruption of the Tfh response to *Plasmodium chabaudi* in C57BL/6 mice and consequent disruption of GC responses and IgG responses and the inability to eliminate the otherwise self-resolving chronic *P. chabaudi* infection. On the other hand, and contrary to previous observations in immunization and viral infection models, Signaling Lymphocyte Activation Molecule (SLAM)-Associated Protein (SAP)-deficient mice were able to activate Tfh cells, GC B cells, and IgG responses to the parasite. This study demonstrates the critical role for Tfh cells in controlling this systemic infection, and highlights differences in the signals required to activate GC B cell responses to this complex parasite compared with those of protein immunizations and viral infections. Therefore, these data are highly pertinent for designing malaria vaccines able to activate broadly protective B-cell responses.

## Highlights

• Chronic *Plasmodium* infection cannot be eliminated in the absence of Tfh cell responses.
• SAP-deficient mice are able to activate GC Tfh and GC B-cell responses to *Plasmodium* infection.
• There is a hierarchical requirement for the control of chronic *Plasmodium* infection following IL-21R > Tfh cells > SAP.

Successful vaccines work through activation of protective B-cell responses. Malaria, caused by *Plasmodium* infection transmitted by mosquito bites, remains a global threat. Despite substantial efforts, a vaccine able to bring about high levels of protection from *Plasmodium* infection remains elusive. Here, using an experimental malaria model including natural mosquito transmission, we demonstrate that proper activation of follicular helper CD4^+^ T cells is essential for the control and eradication of chronic *Plasmodium* infection through protective B-cell responses. Thus, it is strongly advisable for novel vaccine efforts to monitor the robust activation of this important immune compartment.

## 1 Introduction

Follicular helper T cells (Tfh cells) are a particular subpopulation of CD4^+^ T cells that is critically required for the activation of follicular B cells. Since their identification as a discrete CXCR5^+^ subset specialized in collaborating with B cells (Schaerli et al., 2000; Breitfeld et al., 2000; Kim et al., 2001), and the identification of the repressor Bcl6 as the master regulator transcription factor of this subset (Johnston et al., 2009; Nurieva et al., 2009; X. Liu et al., 2009), a large body of research has helped to understand their activation requirements and the differential signals underlying their communication with B cells (Crotty, 2014; Vinuesa et al., 2016). Both soluble factors (i.e. cytokines) and cell surface molecules have been shown to act in parallel to orchestrate this communication. Moreover, several of these signals have been proposed as candidate targets for immunotherapeutic interventions to treat diseases in which B-cell responses are relevant (Hu et al., 2013; Spolski and Leonard, 2014). However, the relative impact of these different types of signals on the outcome of infection or disease remains poorly explored.

The Signaling Lymphocytic Activation Molecule (SLAM)-Associated Protein (SAP) is a small intracellular adaptor protein, which interacts with the cytoplasmic tails of the SLAM family of cell surface receptors and mediates signaling downstream of these receptors (Cannons et al., 2011). Mutations in SAP were originally associated with most cases of X-linked lymphoproliferative syndrome (Cannons et al., 2011). One outstanding function of SAP is to mediate signaling leading to the stable long-duration contact of T and B cells (Qi et al., 2008). This physical interaction between T and B cells, tightly regulated by SAP, has been shown to be critical for the activation of germinal center (GC) B-cells (Qi, 2012), and the activation of Tfh cells in some models (Cannons et al., 2010; Deenick et al., 2010; Linterman et al., 2011).

The B-cell response to the blood stages of *Plasmodium*, the protozoan parasite that causes malaria, is thought to be important for protective immunity in human infections (Cohen et al., 1961; Conway et al., 2000; Fowkes et al., 2010; Osier et al., 2008; Sabchareon et al., 1991). B cells and antibodies are also necessary for the elimination of this stage of infection in experimental mouse models (Burns et al., 1997; von der Weid et al., 1996); thus, these models allow an examination of the relative importance of different Tfh-derived signals in the control of infection. Dysfunctional Tfh responses have been described in children exposed to *P. falciparum*, which are thought to be responsible for impaired development of protective B cell-responses (Obeng-Adjei et al., 2015). *P. berghei* infection in mice inhibits Tfh differentiation (Ryg-Cornejo et al., 2015), whereas boosting of Tfh responses in mice by therapeutic interventions has been shown to accelerate the control of chronic *P. chabaudi* infection (Butler et al., 2012). The critical signals required for Tfh activation to *Plasmodium* infection have also begun to emerge. OX40, PD-1 and ICOS cell surface molecules were shown to regulate Tfh activation during non-lethal *P. yoelii* and *P. chabaudi* infections (Zander et al., 2015; Wikenheiser et al., 2016). We have recently shown that IL-21-producing CD4^+^ T cells, of which a substantial proportion has a Tfh cell phenotype, are required to activate IgG responses to *P. chabaudi* and to control the chronic phase of this infection (Pérez-Mazliah et al., 2015). Interestingly, acute gamma herpes virus co-infection leads to loss of control of an otherwise non-lethal *P. yoelii* infection, and this is associated with a disruption of the Tfh cell response (Matar et al., 2015).

Despite these important advances in our knowledge of Tfh cell responses, a direct link between Tfh cell responses and the control of *Plasmodium* infection remains to be demonstrated, and the relative impact of the different Tfh-derived signals (i.e. cell surface molecular interactions vs soluble factors) on the control of the infection has not been explored in detail. Moreover, despite the substantial differences in infections initiated by artificial versus natural mosquito transmission (Spence et al., 2013), our knowledge of T- and B-cell responses during experimental erythrocytic malaria models has been exclusively generated using artificial injection of infected blood to initiate the infection, thus obviating the full life cycle in the mouse. Here, using both blood transmission as well as a model of natural mosquito transmission, we compared the relative requirements of Tfh responses overall, together with the individual requirements of SAP and IL-21R on the control of *Plasmodium chabaudi* AS infection, a rodent model which presents both an acute and chronic phase (Achtman et al., 2007).

We demonstrate a critical role for Tfh cells in the elimination of the chronic phase of *Plasmodium* infection initiated by both, blood transmission, and natural mosquito transmission. In addition, and contrary to previous observations in immunization studies, and virus and helminth infections (Crotty et al., 2003; Cannons et al., 2006; Kamperschroer et al., 2006; Crotty et al., 2006; McCausland et al., 2007; Moyron-Quiroz et al., 2009; Yusuf et al., 2010; Morra et al., 2005), we show that SAP-deficient mice are able to activate Tfh and GC B cells, and an IgG response to the parasite. Finally, we demonstrate a hierarchy of immune responses needed to control the magnitude of the chronic infection, with IL-21 signaling being the most significant requirement followed by Tfh cells and SAP. Our data demonstrate the need for a fully functioning Tfh response for elimination of blood-stage *Plasmodium* infection, and highlights substantial differences in the signals required to activate Tfh and GC B cell responses to this complex parasite compared to immunizations and other infection models.

## 2 Materials and Methods

### 2.1 Ethical Statements

All scientific experiments involving procedures on mice were approved by the Ethical Review Panel of the MRC National Institute for Medical Research (NIMR). They were performed accordingly to the UK National guidelines of the Animals (Scientific Procedures) Act 1986 under the license reference number PPL 70/8326 authorized and granted by the British Home Office.

### 2.2 Mice

C57BL/6, *Sh2d1a*^*−/−*^ [Sh2d1a^tm1Cpt^ (Wu et al., 2001), RRID:MGI:3576735], *CD4-Cre*^*+/−*^ [Tg(Cd4-cre)1Cwi (P. P. Lee et al., 2001), RRID:MGI:3691126], *Bcl6*^*fl/fl*^ [Bcl6^tm1.1Mtto^ (Kaji et al., 2012)], *CD4-Cre*^*+/−*^* Bcl6*^*fl/fl*^ (RRID:MGI:5461330) and *Rag2*^*−/−*^ [Rag2^tm1Fwa^ (Shinkai et al., 1992), RRID:MGI:3617415] mouse strains were bred in the specific pathogen-free facilities of the Mill Hill Laboratory of the Francis Crick Institute, and were backcrossed for at least 10 generations onto NIMR C57BL/6 mice. For experimental use, 6–12 weeks old female mice were housed in conventional facilities with sterile bedding, food and water under reversed light conditions (dark: 7.00 h to 19.00 h).

### 2.3 Infections

*Plasmodium chabaudi chabaudi* (AS) was originally obtained from David Walliker, University of Edinburgh. Infections were initiated by intraperitoneal injection of 10^5^ infected red blood cells, or by the bites of infected *A. stephensi* mosquitoes as previously described (Spence et al., 2012). Blood-stage parasitemias were monitored by Giemsa-stained thin blood smears (Langhorne et al., 1989).

### 2.4 Flow Cytometry

Spleens were dissected and mashed through 70 μm filter mesh in Hank's Balanced Salt Solution (HBSS, Gibco, Invitrogen) to generate single cell suspensions. Spleens were treated in RBC lysis buffer (Sigma) and remaining cells resuspended in complete Iscove's Modified Dulbecco's Medium (IMDM supplemented with 10% fetal bovine serum (FBS) Serum Gold (PAA Laboratories, GE Healthcare), 2 mM l-glutamine, 0.5 mM sodium pyruvate, 100 U penicillin, 100 mg streptomycin, 6 mM Hepes buffer, and 50 mM 2-ME (all from Gibco, Invitrogen). Viable cells were counted based on trypan blue (Sigma) exclusion in a hemocytometer. 2 × 10^6^ viable cells were distributed to each well of a 96-well plate (Nunc) and incubated in the presence of a monoclonal anti-mouse CD16/32 antibody (Unkeless and Unkeless, 1979, BioLegend Cat# 101318 RRID:AB_2104156) to block Fc-mediated binding of antibodies for 20 min at 37 °C, 20 min at 4 °C.

To identify Tfh cells, cells were first incubated with biotin anti-CXCR5 (BD Biosciences Cat# 551960 RRID:AB_394301) in complete IMDM (BD Pharmingen), washed twice with a staining buffer (1 × PBS (pH = 7.2–7.4), 2% FBS, 0.01% Sodium azide), resuspended in 1 × PBS and incubated with appropriate dilutions of PE streptavidin, APC-Cy7 anti-CD4 (BioLegend Cat# 100414 RRID:AB_312699), PE-Cy7 anti-PD-1 (BioLegend Cat# 109110 RRID:AB_572017), Pacific Blue anti-CD3, PerCP-Cy5.5 or FITC anti-CD44 (BioLegend) for 30–40 min at 4 °C. A commercial kit (eBioscience) was used for intra-nuclear detection of Bcl6, following manufacturer's instructions, and using the anti-human and mouse Bcl-6 antibody conjugated to Alexa Fluor 647 (BD Biosciences Cat# 561525 RRID:AB_10898007).

To identify germinal center Tfh and B cells, spleen cells were incubated with appropriate dilutions of PerCP-Cy5.5 anti-CD3 (BioLegend Cat# 100328 RRID:AB_893318), PE anti-CD4 (BioLegend Cat# 100408 RRID:AB_312693), PE-Cy7 anti-CD45R/B220 (BioLegend Cat# 103222 RRID:AB_313005), APC-Cy7 anti-CD19 (BioLegend Cat# 115530 RRID:AB_830707), APC anti-CD38 (BioLegend Cat# 102712 RRID:AB_312933) FITC anti-GL-7 (BioLegend Cat# 144604 RRID:AB_2561697) and BV421 anti-IgD (BioLegend Cat# 405725 RRID:AB_2562743) for 30–40 min at 4 °C. Germinal center B cells were identified by combined expression of CD19 and B220, low expression of IgD and CD38, and high expression of GL-7. Tfh cells were identified by the combined expression of CD3, CD4 and GL-7. T-B cell conjugates were studied ex vivo by flow cytometry as previously described (Reinhardt et al., 2009). Conjugates were identified by the combined expression of CD3, CD4, CD19 and B220, and identified as doublets based on FSC-A vs FSC-W.

IL-21 detection by flow cytometry was done by intracellular staining as previously described (Pérez-Mazliah et al., 2015).

All cells were fixed with 2% paraformaldehyde for 15 min at 4 °C, washed and stored in the staining buffer at 4 °C until acquisition. Cells were acquired on BD FACSVerse, BD LSRII or BD LSRFortessa X-20 (BD Biosciences) flow cytometers. Dead cells were excluded by staining with LIVE/DEAD Aqua (Invitrogen) prior fixation. Fluorescence-minus-one (FMO) controls were used to set the thresholds for positive/negative events. Analysis was performed with FlowJo software version 9.6 or higher (Tree Star). Doublets based on side scatter light and forward scatter light Area vs Width were routinely excluded from the analysis, with the exemption of the assay to study T-B cell conjugates, in which doublets were deliberated included in the analysis.

### 2.5 Histology

Spleens were dissected and fixed for 24 h in 10% neutral buffered formalin (Sigma), embedded in wax, sectioned (4 μm), and stained by incubating with rat Anti-mouse CD45R/B220 (BD Biosciences Cat# 550286 RRID:AB_393581), rabbit anti CD3G (Abcam Cat# ab108996 RRID:AB_10862885), biotinylated peanut agglutinin (PNA, Vector Laboratories Cat# B-1075 RRID:AB_2313597), followed by incubation with goat anti-rat IgG alexa fluor 647 (Molecular Probes Cat# A-21247 also A21247 RRID:AB_141778), goat anti-rabbit IgG alexa fluor 488 (Molecular Probes Cat# A-11008 also A11008 RRID:AB_143165), streptavidin alexa fluor 555 (ThermoFisher), and DAPI (ThermoFisher). Stained sections were scanned on a ZEISS Axio Scan.Z1 with ZEN 2 software (Zeiss). Area, perimeter and number of PNA^+^ GC were determined with Fiji (Schindelin et al., 2012).

### 2.6 *P. chabaudi*-specific ELISAs

Serum samples were obtained periodically after *P. chabaudi* infection by bleeding the mice from the tail vein. Whole parasite lysate was generated and used in ELISAs to measure specific IgM, IgG and IgG subclasses, as previously described (Quin and Langhorne, 2001), with the SBA Clonotyping System-C57BL/6-HRP (SouthernBiotech Cat# 5300-05B). A pool of plasma from NIMR C57BL/6 mice challenged at least six times with *P. chabaudi* was used as a standard for each isotype, and defined as 1000 arbitrary units (AU). The amounts of each isotype were calculated in AU derived from this standard, which was run in parallel with every experimental sample assayed.

### 2.7 Statistical Analysis

Statistical analyses were performed using Mann Whitney *U* test, Kruskal-Wallis test with Dunn's multiple comparisons test, two-way ANOVA with Tukey's multiple comparisons test, or Chi-square test on Prism v7 (GraphPad). P < 0.05 was accepted as a statistically significant difference.

## 3 Results

### 3.1 *Bcl6*^*fl/fl*^ and SAP-deficient Mice; Two Different Models to Explore the Role of Tfh Responses During *P. chabaudi* Infection

The repressor *Bcl6* has been identified as the master transcription factor regulating the Tfh cell program (Johnston et al., 2009; Nurieva et al., 2009; X. Liu et al., 2009). In addition, SAP (encoded by the *Sh2d1a* gene) has been shown to signal directly in CD4^+^ T cells and to regulate Tfh cell activation in some immunization models (Cannons et al., 2010; Deenick et al., 2010; Linterman et al., 2011). We therefore explored the effects of inactivation of the *Bcl6* gene in T cells [*Bcl6*^*fl/fl*^*CD4-cre*^*+/−*^ (Kaji et al., 2012)] or the *Sh2d1a gene* [*Sh2d1a*^*−/−*^ (Wu et al., 2001), encoding SAP] on the ability to control a chronic *P. chabaudi* blood-stage infection.

**Fig. 1:**
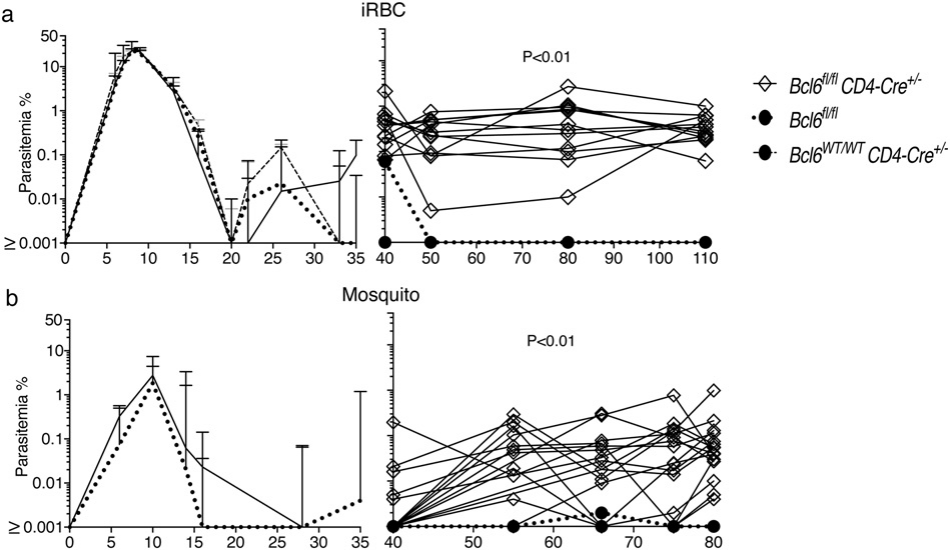
Bcl6 expression on T cells is required for the control of chronic *P. chabaudi* infection. *Bcl6*^*fl/fl*^*CD4-cre*^*+/−*^ and their respective *wt* control mice were infected with *P. chabaudi* via intra peritoneal injection of 10^5^ iRBC (a), or bites from *P. chabaudi*-infected mosquitoes (b), and parasitemias were periodically monitored by giemsa-stained thin blood smears. The course of acute (left column) and chronic (right column) infection is displayed. The acute phase is displayed as median + range, while curves corresponding to individual mice are displayed for the chronic phase. Data are representative of at least two independent experiments and were obtained in groups of 5–16 mice per time point. Statistical significance was obtained using Mann Whitney *U* test or Kruskal-Wallis test.

*Bcl6*^*fl/fl*^*CD4-cre*^*+/−*^ mice, deficient in Tfh cell responses to immunizations (Kaji et al., 2012), showed no alteration in the control of the acute phase of infection up to day 25, compared with infections in *Bcl6*^*wt/wt*^*CD4-cre*^*+/−*^ and *Bcl6*^*fl/fl*^ wild-type (*wt*) control mice (Fig. 1a). Acute stage parasitemias were reduced to 0.05% or lower in the *Bcl6*^*fl/fl*^*CD4-cre*^*+/−*^ as well as in their respective *wt* control mice. However, *Bcl6*^*fl/fl*^*CD4-cre*^*+/−*^ mice failed to control or eradicate the chronic phase of infection, and showed parasitemias variably around 1% iRBC for the duration of the experiment (parasitemias of individual mice are shown in right hand graph of Fig. 1a). During this time, *wt* mice had reduced parasitemias to < 0.001% (i.e. below detection limit, Fig. 1a). Importantly, similar patterns of acute and chronic infection were also observed in *Bcl6*^*fl/fl*^*CD4-cre*^*+/−*^ mice infected via mosquito bites rather than by direct injection of iRBC (Fig. 1b).

**Fig. 2:**
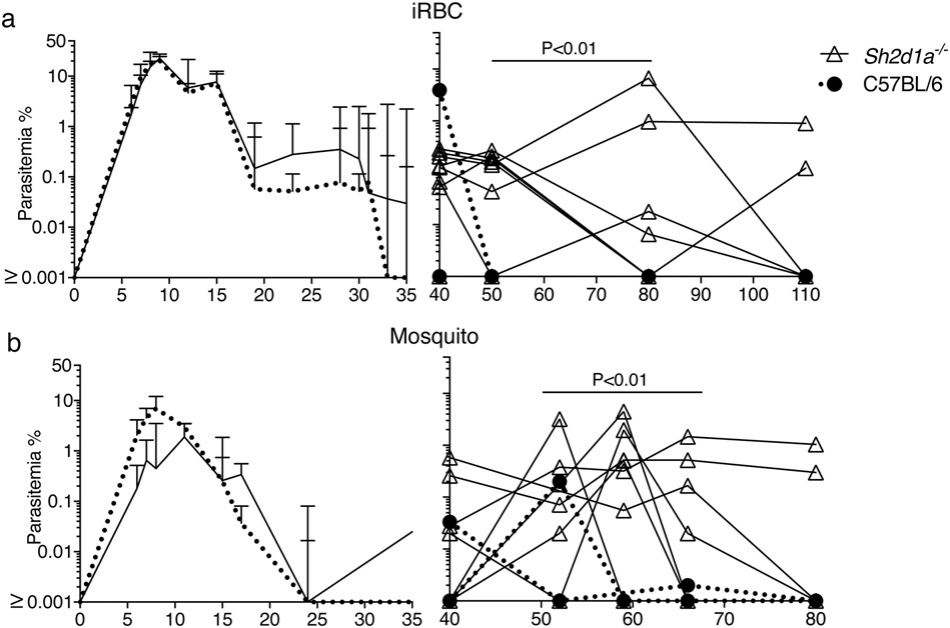
Control of chronic *P. chabaudi* is altered by the absence of SAP signaling. *Sh2d1a*^*−/−*^ and C57BL/6 *wt* control mice were infected with *P. chabaudi* via intra peritoneal injection of 10^5^ iRBC (a), or bites from *P. chabaudi*-infected mosquitoes (b), and parasitemias were periodically monitored by giemsa-stained thin blood smears. The course of acute (left column) and chronic (right column) infection is displayed. The acute phase is displayed as median + range, while curves corresponding to individual mice are displayed for the chronic phase. Data are representative of at least two independent experiments and were obtained in groups of 7–8 mice per time point. Statistical significance was obtained using Mann Whitney U test.

The lack of SAP had a more moderate, yet detectable, effect on the *P. chabaudi* infection. The acute infection in both *Sh2d1a*^*−/−*^ and *wt* C57BL/6 mice was similarly reduced to < 0.1% by day 18 (Fig. 2a). Although chronic parasitemias were initially higher in all *Sh2d1a*^*−/−*^ mice than in *wt* mice, parasitemias were very variable between individual mice. Eventually by 110 days p.i. all *wt* C57BL/6 mice had undetectable parasitemias (i.e. < 0.001%), and only 2/8 of the *Sh2d1a*^*−/−*^ mice showed detectable parasitemias (Fig. 2a), suggesting clearance of infection in all *wt* controls and the majority of *Sh2d1a*^*−/−*^ mice. However, a technique more sensitive to detect parasitemia, i.e. transfer of 100 μl of blood from *Sh2d1a*^*−/−*^ and *wt* mice at this time into *Rag2*^*−/−*^ mice, demonstrated that 50% of these *Sh2d1a*^*−/−*^ mice were still sub-patently infected, whereas confirming that all *wt* C57BL/6 mice had cleared their infection (Table 1). Thus, these data demonstrate that although there is significant control of the chronic infection, SAP signaling is required for the complete elimination of the *P. chabaudi* infection. Importantly, a similar pattern of chronic infection was observed in mice infected via mosquito bites rather than by direct injection of iRBC (Fig. 2b).

**Table 1:** Number of parasitemic C57BL/6 and *Sh2da1*^*−/−*^ blood donors (> 100 p.i.), and parasitemic *Rag2*^*−/−*^ blood recipients.

	Parasitemic donor (before blood transference)^a^	Parasitemic *Rag2*^*−/−*^ recipient (after blood transference)^b^
C57BL/6	0/8 (0%)	0/8 (0%)
*Sh2da1*^*−/−*^	2/8 (25%)	4/8 (50%)

a χ^2^, P > 0.05 (ns).

b χ^2^, P < 0.05.

Altogether, these data demonstrate that the inactivation of the *Bcl6* gene in T cells, encoding the Tfh program, has a dramatic effect in the control of chronic *Plasmodium* infection, and unexpectedly more pronounced compared to the lack of SAP signaling.

### 3.2 Bcl6, but not SAP, is Required for the Initial Tfh Cell Response to *P. chabaudi* Infection

**Fig. 3:**
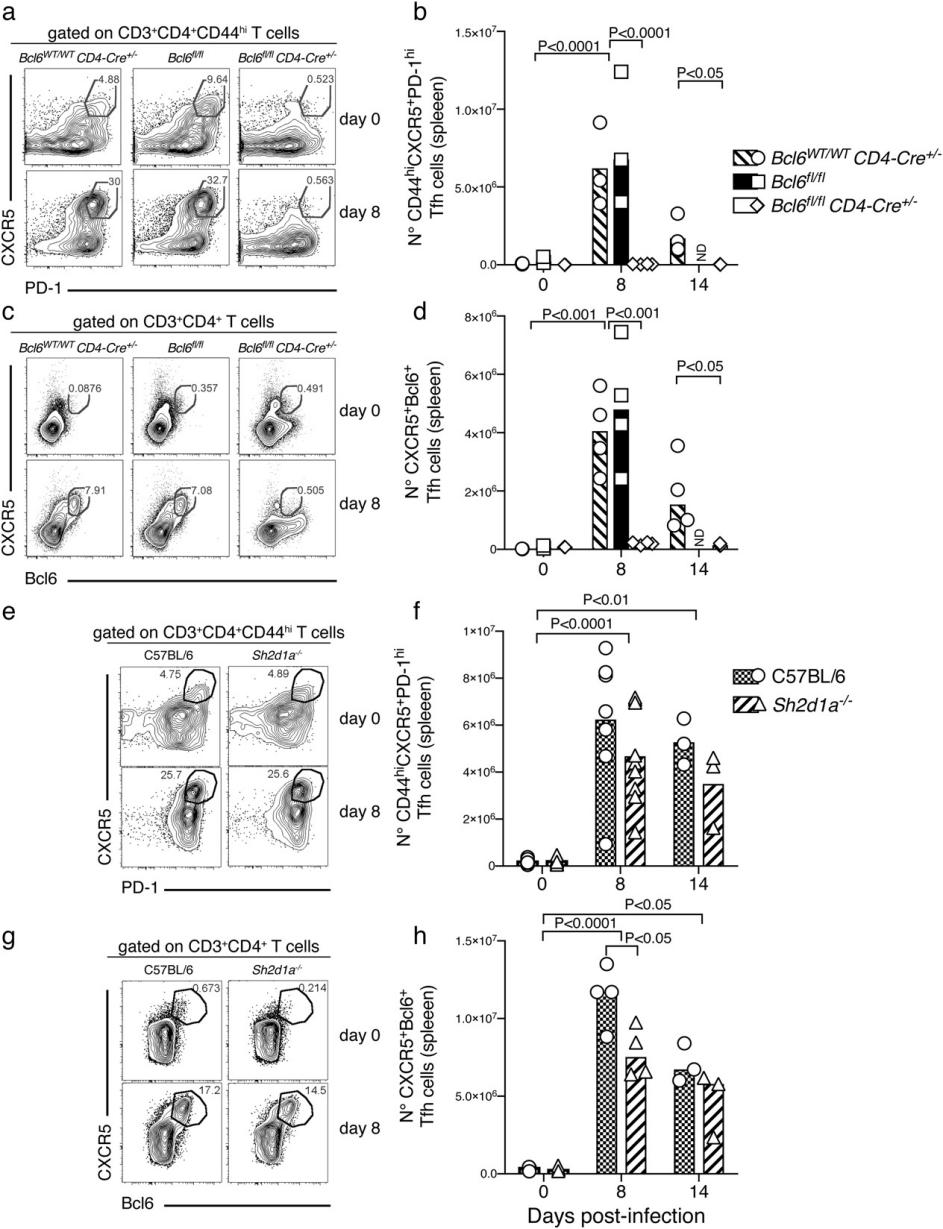
Tfh cell responses in *P. chabaudi* infection are abrogated in *Bcl6*^*fl/fl*^*CD4-cre*^*+/−*^ but not in SAP-deficient mice. *Bcl6*^*fl/fl*^*CD4-cre*^*+/*^^*−*^ and *Sh2d1a*^*−/−*^ mice were infected with *P. chabaudi* via intra peritoneal injection of 10^5^ iRBC, and the occurrence of Tfh cells in the spleen was determined by flow cytometry at days 0, 8 and 14 post-infection. (a) Representative plots of naïve (top row) and infected (8 days post-infection, bottom row) *Bcl6*^*fl/fl*^*CD4-cre*^*+/*^^*−*^ and *wt* control mice. Gates show frequency of CD3^+^ CD4^+^ CD44^hi^ cells expressing CXCR5 and PD-1. (b) Total numbers of Tfh cells, defined as CD3^+^ CD4^+^ CD44^hi^CXCR5^hi^PD-1^hi^, in *Bcl6*^*fl/fl*^*CD4-cre*^*+/*^^*−*^ and *wt* control mice. (c) Representative plots of naïve (top row) and infected (8 days post-infection, bottom row) *Bcl6*^*fl/fl*^*CD4-cre*^*+/*^^*−*^ and *wt* control mice. Gates show frequency of CD3^+^ CD4^+^ cells expressing CXCR5 and Bcl6. (d) Total numbers of Tfh cells, defined as CD3^+^ CD4^+^ CXCR5^+^ Bcl6^+^, in *Bcl6*^*fl/fl*^*CD4-cre*^*+/*^^*−*^ and *wt* control mice. (e) Representative plots of naïve and infected *Sh2d1a*^*−/−*^ and *wt* C57BL/6 control mice. (f) Total numbers of Tfh cells, defined as CD3^+^ CD4^+^ CD44^hi^CXCR5^hi^PD-1^hi^, in *Sh2d1a*^*−/−*^ and *wt* C57BL/6 control mice. (g) Representative plots of naïve and infected *Sh2d1a*^*−/−*^ and *wt* C57BL/6 control mice. (h) Total numbers of Tfh cells, defined as CD3^+^ CD4^+^ CXCR5^+^ Bcl6^+^, in *Sh2d1a*^*−/−*^ and *wt* C57BL/6 control mice. Bars represent median values. Data are representative or pooled from at least two independent experiments and were obtained in groups of 3–8 mice per time point. Statistical significance was obtained using Two-Way ANOVA with Tukey's multiple comparisons test. ND: Not done.

As both Bcl6 and SAP have been shown to be required for fully functional Tfh responses, we then studied the activation of Tfh cell responses to *P. chabaudi* in *Bcl6*^*fl/fl*^*CD4-cre*^*+/−*^ and *Sh2d1a*^*−/−*^ mice. We have previously shown that the Tfh cell response to *P. chabaudi* peaks around days 7–9 post-infection in the spleen of *wt* C57BL/6 mice (Pérez-Mazliah et al., 2015). Therefore, Tfh cells were enumerated in the spleen at 0, 8 and 14 days post-infection in *Bcl6*^*fl/fl*^*CD4-cre*^*+/−*^, *Sh2d1a*^*−/−*^ and *wt* control mice (Fig. 3).

**Supplementary Fig. S1:**
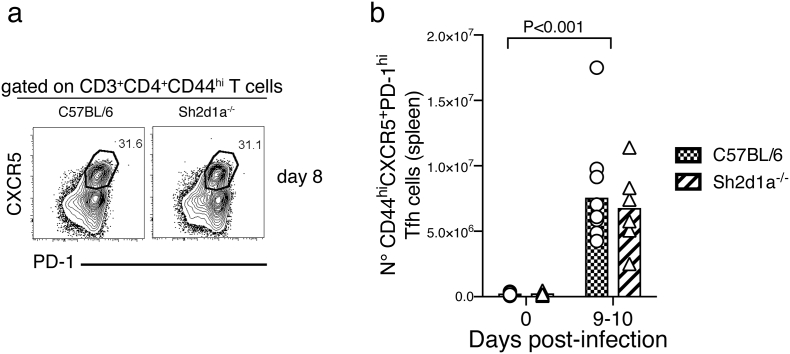
Related to Fig. 3. Normal Tfh cell activation to *P. chabaudi* in SAP-deficient mice infected with *P. chabaudi* via natural mosquito transmission. *Sh2d1a*^*−/−*^ and C57BL/6 *wt* control mice were infected with *P. chabaudi* via mosquito bites, and the activation of Tfh cells in the spleen was determined by flow cytometry. (a) Representative plots of C57BL/6 *wt* control (left) and *Sh2d1a*^*−/−*^ (right) mice, 8 days post-infection. Gates show frequency of CD3^+^ CD4^+^ CD44^hi^ cells expressing CXCR5 and PD-1. (b) Total numbers of Tfh cells, defined as CD3^+^ CD4^+^ CD44^hi^CXCR5^hi^PD-1^hi^, in *Sh2d1a*^*−/−*^ and C57BL/6 *wt* control mice. Bars represent median values. Data are representative of two independent experiments and were obtained in groups of 5–8 mice per time point. Statistical significance was obtained using Two-Way ANOVA with Tukey's multiple comparisons test.

As expected, targeted deletion of the *Bcl6* gene in T cells completely abrogated Tfh cell activation in response to *P. chabaudi*; no CD44^high^CXCR5^+^ PD-1^high^ Tfh cells were detected above background levels in the spleen of *Bcl6*^*fl/fl*^*CD4-cre*^*+/*^^*−*^ mice during *P. chabaudi* infection (Fig. 3a–b). Tfh cells can be alternatively identified by the combined expression of CXCR5 and Bcl6 (Choi et al., 2011). Using this alternative phenotypic definition of Tfh, we confirmed no expansion of CXCR5^+^ Bcl6^+^ Tfh cells in *Bcl6*^*fl/fl*^*CD4-cre*^*+/*^^*−*^ mice during *P. chabaudi* infection (Fig. 3 c–d). In contrast to *Bcl6*^*fl/fl*^*CD4-cre*^*+/−*^ mice, *Sh2d1a*^*−/−*^ mice showed no major alterations in the activation of Tfh cell responses to *P. chabaudi* infection. These mice showed an expansion of CD44^high^CXCR5^+^ PD-1^high^ Tfh cells (Fig. 3 e–f) and CXCR5^+^ Bcl6^+^ Tfh cells (Fig. 3 g–h) in response to *P. chabaudi* infection, comparable to the levels detected in *wt* C57BL/6 control mice [we only detected a small, yet significant, reduction of the counts of CXCR5^+^ Bcl6^+^ cells in *Sh2d1a*^*−/−*^ mice at day 8 post infection (Fig. 3H)]. This was independent of the route of infection, as *Sh2d1a*^*−/−*^ mice infected via mosquito bites also activated a CD44^high^CXCR5^+^ PD-1^high^ Tfh cell response comparable to the levels detected in similarly infected *wt* C57BL/6 control mice (Fig. S1).

**Supplementary Fig. S2:**
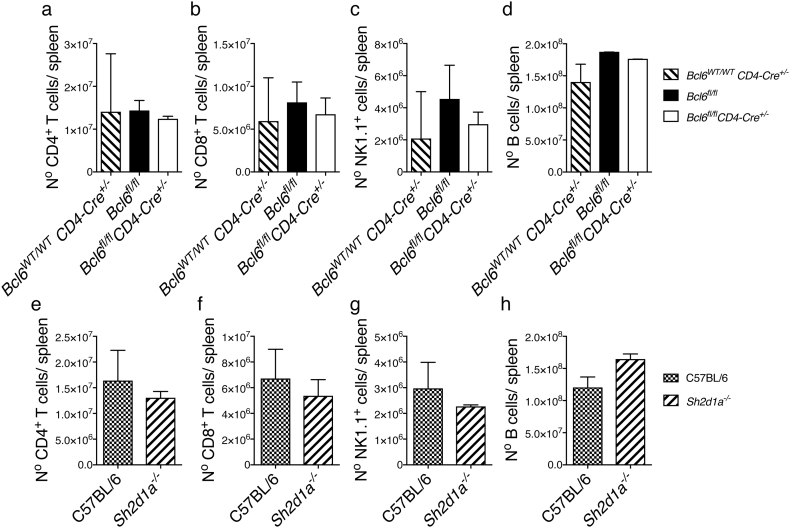
Related to Fig. 3. Normal T, B and NK cell development in the spleen of *Bcl6*^*fl/fl*^*CD4-cre*^*+/−*^ and SAP-deficient mice. Total numbers of CD4 T (a–e), CD8 T (b–f), NK (c–g) and B (d–h) cells in the spleen of *Bcl6*^*fl/fl*^*CD4-cre*^*+/−*^ and *wt* control mice (top), and *Sh2d1a*^*−/−*^ and C57BL/6 *wt* control mice (bottom). Bars show median ± range. Data are pooled from two independent experiments and were obtained in groups of 3–5 mice. Statistical significance was evaluated using Mann Whitney *U* test or Kruskal-Wallis test.

**Supplementary Fig. S3:**
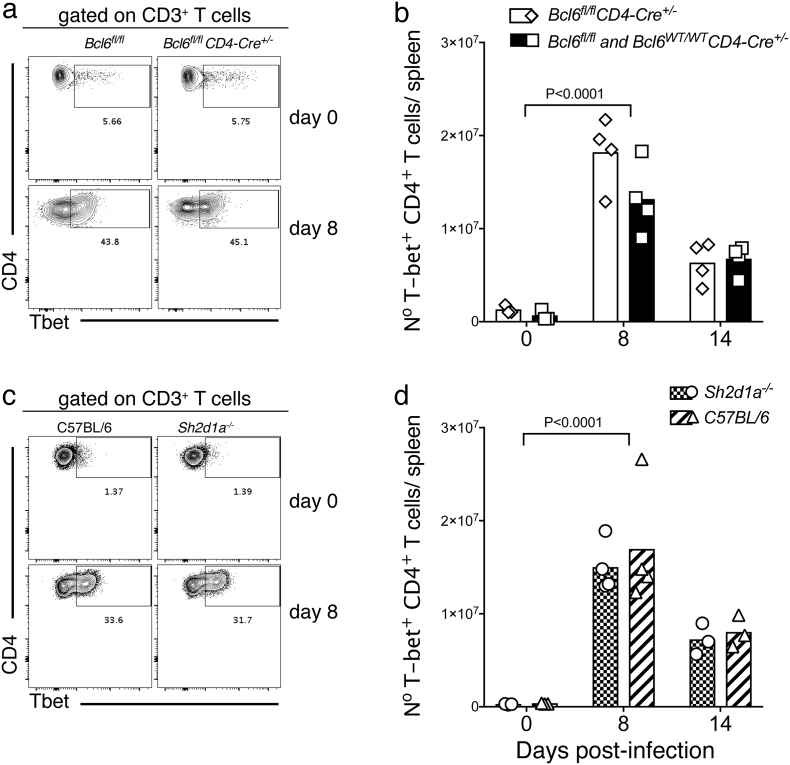
Related to Fig. 3. Normal activation of Th1 cell responses in the spleen of *Bcl6*^*fl/fl*^*CD4-cre*^*+/−*^ and SAP-deficient mice. *Bcl6*^*fl/fl*^*CD4-cre*^*+/*^^*−*^ and *Sh2d1a*^*−/−*^ mice were infected with *P. chabaudi* via intra peritoneal injection of 10^5^ iRBC, and the occurrence of T-bet^+^ CD4^+^ Th1 cells in the spleen was determined by intracellular flow cytometry staining at days 0, 8 and 14 post-infection. (a) Representative plots of naïve (top row) and infected (8 days post-infection, bottom row) *Bcl6*^*fl/fl*^*CD4-cre*^*+/−*^ and *wt* control mice. Gates show frequency of CD3^+^ CD4^+^ T-bet^+^ cells. (b) Total numbers of CD3^+^ CD4^+^ T-bet^+^ Th1 cells in *Bcl6*^*fl/fl*^*CD4-cre*^*+/−*^ and *wt* control mice. (c) Representative plots of naïve and infected *Sh2d1a*^*−/−*^ and *wt* C57BL/6 control mice. (d) Total numbers of CD3^+^ CD4^+^ T-bet^+^ Th1 cells in *Sh2d1a*^*−/−*^ and *wt* C57BL/6 control mice. Bars represent median values. Data are representative of two independent experiments and were obtained in groups of 3–4 mice per time point. Statistical significance was obtained using Two-Way ANOVA with Tukey's multiple comparisons test.

In agreement with previous publications (Wu et al., 2001; Czar et al., 2001; Kaji et al., 2012), neither *Bcl6*^*fl/fl*^*CD4-cre*^*+/−*^, nor *Sh2d1a*^*−/−*^ mouse strains showed alterations in the counts of CD4^+^ and CD8^+^ T cells, B cells and NK cells in the spleen prior to infection (Fig. S2). In addition, neither *Bcl6*^*fl/fl*^*CD4-cre*^*+/−*^, nor *Sh2d1a*^*−/−*^ mouse strains showed significant alterations in the activation of T-bet^+^ CD4^+^ Th1 responses during this strong Th1-biased *P. chabaudi* infection (Fig. S3).

These data demonstrate that the initial Tfh cell response to *P. chabaudi* can be activated even in the absence of SAP signaling, in contrast to infected *Bcl6*^*fl/fl*^*CD4-cre*^*+*^ mice, in which Tfh cells were lacking.

### 3.3 The Lack of Tfh Cells and Deficiency of SAP Differentially Impact the Activation of GC B-cells in Response to *P. chabaudi* Infection

**Fig. 4:**
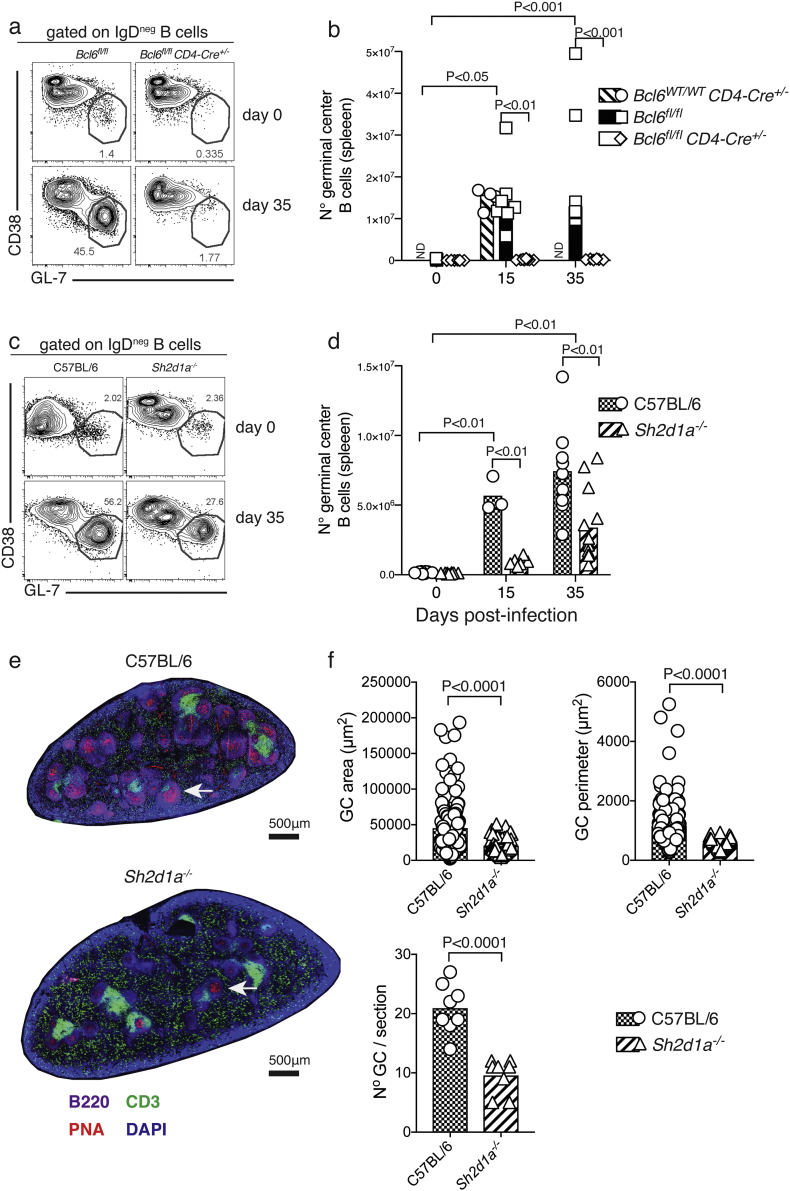
Germinal Center B-cell responses to *P. chabaudi* infection are abrogated in *Bcl6*^*fl/fl*^*CD4-cre*^*+/−*^ mice, and reduced in SAP-deficient mice. *Bcl6*^*fl/fl*^*CD4-cre*^*+/*^^*−*^ and *Sh2d1a*^*−/−*^ mice were infected with *P. chabaudi* via intra peritoneal injection of 10^5^ iRBC, and the occurrence of GC B cells in the spleen was determined by flow cytometry at days 0, 15 and 35 post-infection, and by histology at days 0 and 35 post-infection. (a) Representative plots of naïve (top row) and infected (35 days post-infection, bottom row) *Bcl6*^*fl/fl*^*CD4-cre*^*+/*^^*−*^ and *wt* control mice. Gates show frequency of B220^+^ CD19^+^ IgD^neg^ B cells expressing low levels of CD38 and high levels of GL-7. (b) Total numbers of GC B cells in *Bcl6*^*fl/fl*^*CD4-cre*^*+/−*^ and *wt* control mice. (c) Representative plots of naïve and infected *Sh2d1a*^*−/−*^ and *wt* C57BL/6 control mice. (d) Total numbers of GC B cells in *Sh2d1a*^*−/−*^ and *wt* C57BL/6 control mice. Bars represent median values. (e) Representative spleen sections of infected (d35) *Sh2d1a*^*−/−*^ and *wt* C57BL/6 control mice; the white arrows point at individual examples of PNA^+^ GC structures. (f) Cumulative data displaying area, perimeter and number of GC structures determined by histology in spleen sections of infected (d35) *Sh2d1a*^*−/−*^ and *wt* C57BL/6 control mice. Data are representative or pooled from at least two independent experiments and were obtained in groups of 3–8 mice per time point. Statistical significance was obtained using Student's *t*-test or Two-Way ANOVA with Tukey's multiple comparisons test. ND: Not done.

We have previously shown that robust GC B-cell responses to *P. chabaudi* infection in the spleen of C57BL/6 mice are readily detected at days 14–40 p.i. (Pérez-Mazliah et al., 2015; Achtman et al., 2003). In the absence of Tfh-cell responses, *Bcl6*^*fl/fl*^*CD4-cre*^*+/−*^ mice were unable to activate a GC B-cell response to *P. chabaudi* infection, while robust GC B-cell responses were detected in both *Bcl6*^*wt/wt*^*CD4-cre*^*+/−*^ and *Bcl6*^*fl/fl*^ control mice by day 35 p.i, as determined by flow cytometry (Fig. 4 a–b). It has previously been shown that SAP-deficient mice are unable to activate GC-B cell responses to protein antigens and Influenza virus, LCMV and helminth infections (Crotty et al., 2003; Cannons et al., 2006; Kamperschroer et al., 2006; Crotty et al., 2006; McCausland et al., 2007; Moyron-Quiroz et al., 2009; Yusuf et al., 2010; Morra et al., 2005). Surprisingly, although the number of GC B-cells activated in the spleen of infected SAP-deficient mice was reduced compared with *wt* C57BL/6 controls, these mice were still able to activate a distinctive GC B-cell response to *P. chabaudi* infection, readily detectable by flow cytometry by day 35 p.i. (Fig. 4 c–d). In accordance, GC structures were readily detected in spleen sections of SAP-deficient mice by day 35 p.i. (Fig. 4 E), although at reduced numbers and size compared with *wt* C57BL/6 controls (Fig. 4 F).

These data show that GC B-cell responses take place in the absence of SAP, and highlight substantial differences in the signaling network leading to GC activation in response to *Plasmodium* compared with GC responses after immunizations or in other infection models.

### 3.4 The GC Tfh Cell Responses to *P. chabaudi* are Affected, but not Entirely Abrogated, in the Absence of SAP Signaling

The activation of a particular subset of CD4^+^ Tfh cells which homes into germinal centers (GC Tfh), has been shown to follow the initial activation of Tfh-cell responses, to express the germinal center marker GL-7, to be critical for GC B-cell activation, and to require SAP signaling for its activation (Yusuf et al., 2010). We therefore examined the activation of GC Tfh cells in the spleen during *P. chabaudi* infection.

**Fig. 5:**
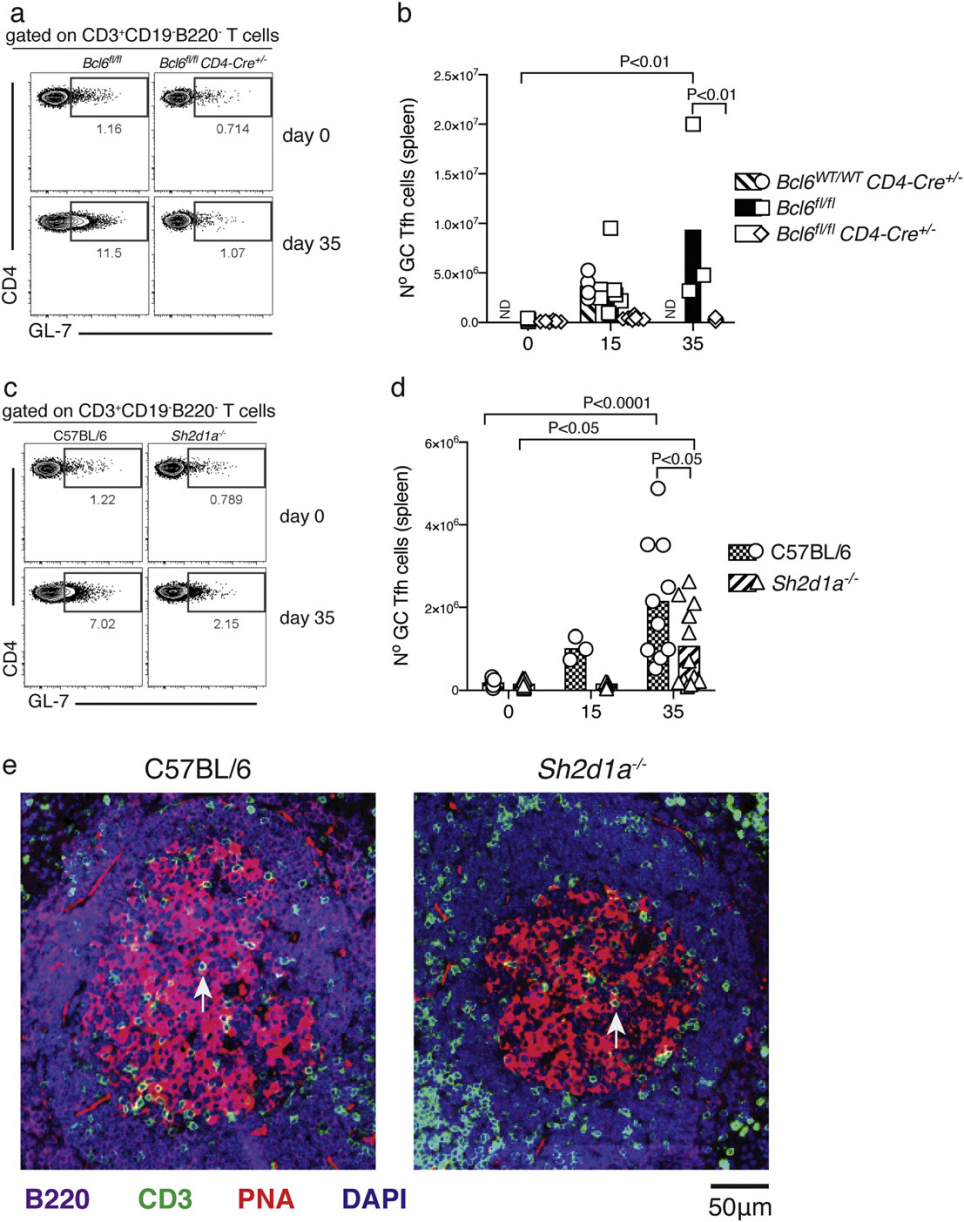
Germinal center Tfh-cell responses to *P. chabaudi* infection are abrogated in *Bcl6*^*fl/fl*^*CD4-cre*^*+/−*^ mice, and reduced in SAP-deficient mice. *Bcl6*^*fl/fl*^*CD4-cre*^*+/*^^*−*^ and *Sh2d1a*^*−/−*^ mice were infected with *P. chabaudi* via intra peritoneal injection of 10^5^ iRBC, and the occurrence of GC Tfh cells in the spleen defined as CD3^+^ CD4^+^ GL-7^+^ was determined by flow cytometry at days 15 and 35 post-infection. (a) Representative plots of naïve (top row) and infected (35 days post-infection, bottom row) *Bcl6*^*fl/fl*^*CD4-cre*^*+/*^^*−*^ and *wt* control mice. Gates show frequency of CD3^+^ CD4^+^ T cells expressing GL-7. (b) Total numbers of GC B cells in *Bcl6*^*fl/fl*^*CD4-cre*^*+/−*^ and *wt* control mice. (c) Representative plots of naïve and infected *Sh2d1a*^*−/−*^ and *wt* C57BL/6 control mice. (d) Total numbers of Tfh cells in *Sh2d1a*^*−/−*^ and *wt* C57BL/6 control mice. Bars represent median values. (e) Representative spleen sections of infected (d35) *Sh2d1a*^*−/−*^ and *wt* C57BL/6 control mice; the white arrows point at individual examples of CD3^+^ T cells within PNA^+^ GC structures. Data are representative or pooled from at least two independent experiments and were obtained in groups of 3–8 mice per time point. Statistical significance was obtained using Two-Way ANOVA with Tukey's multiple comparisons test. ND: Not done.

As anticipated by the absence of early Tfh responses to *P. chabaudi* infection in *Bcl6*^*fl/fl*^*CD4-cre*^*+/−*^ mice, no GC Tfh cells were detected above background level at any time point post *P. chabaudi* infection in these mice (Fig. 5 a–b). On the other hand, although reduced compared to *wt* C57BL/6 controls, a GC Tfh cell response to *P. chabaudi* was readily detected by flow cytometry by day 35 p.i. in *Sh2d1a*^*−/−*^ mice (Fig. 5 c–d). The occurrence of GC Tfh cells in the absence of SAP was further confirmed by the detection of T cells inside the GC structures in the spleen, as determined by histology (Fig. 5 e–f).

These data demonstrate that, although the initial activation of Tfh-cell responses to *P. chabaudi* showed no major alterations in SAP-deficient mice, this signal is indeed required later in the infection for full activation of a GC Tfh-cell response, and further support a requirement for fully functional Tfh-dependent B-cell activation to eradicate *Plasmodium* infection.

### 3.5 The B-T Cell Interactions in the Spleen are Affected, but not Entirely Abrogated, in the Absence of SAP Signaling During *P. chabaudi* Infection

SAP signaling is required for stable B-Tfh cell interactions, which are thought to be necessary for activation of GC B-cell responses (Qi, 2012). Given the unexpected presence of GC Tfh and GC B cells in the absence of SAP signaling in the *P. chabaudi* infection, we asked whether stable B-T cell conjugates could form in the spleen during *P. chabaudi* infection.

**Fig. 6:**
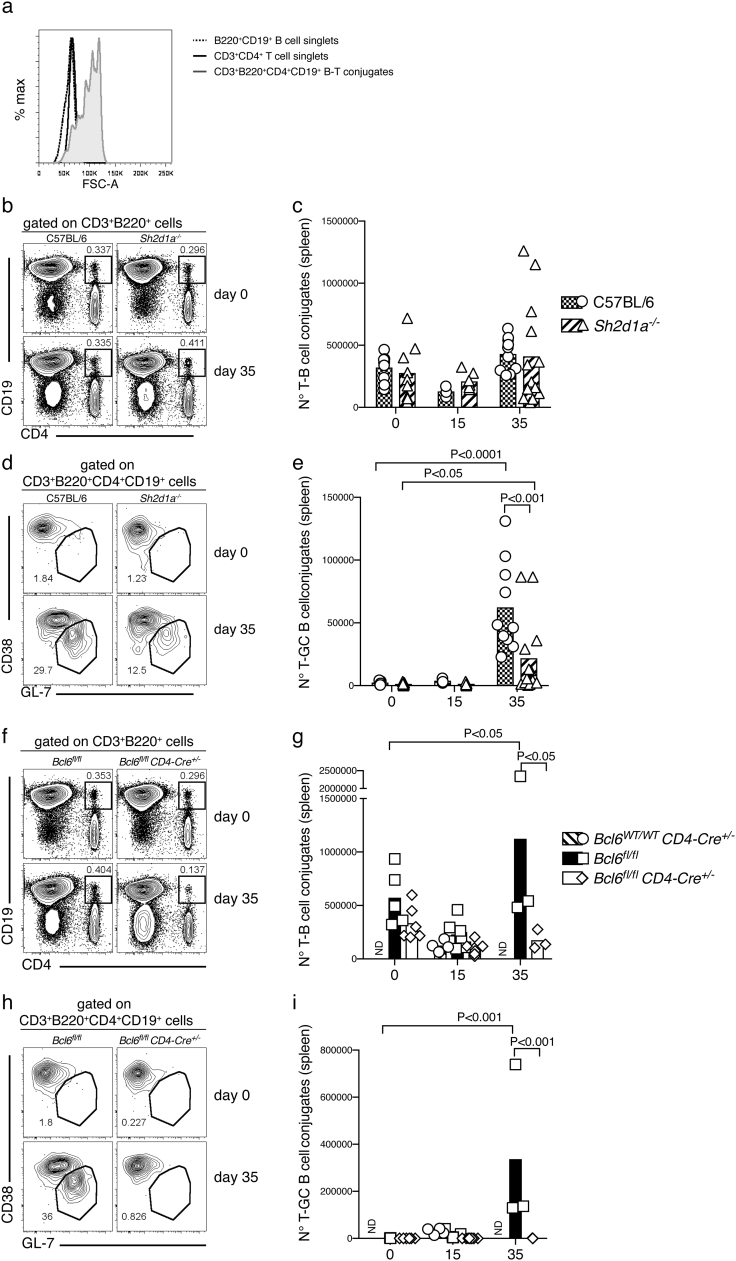
Ex vivo flow cytometry analysis of T-B cell conjugates in *Bcl6*^*fl/fl*^*CD4-cre*^*+/−*^ and SAP-deficient mice during *P. chabaudi* infection. *Bcl6*^*fl/fl*^*CD4-cre*^*+/*^^*−*^ and *Sh2d1a*^*−/−*^ mice were infected with *P. chabaudi* via intra peritoneal injection of 10^5^ iRBC, and the occurrence of T-B cell conjugate in the spleen was analyzed ex vivo by flow cytometry at days 0, 15 and 35 post-infection. (a) Representative overlay histograms showing FSC-A for CD3^+^ CD4^+^ T cell singlets, B220^+^ CD19^+^ B cell singlets, and CD3^+^ CD4^+^ B220^+^ CD19^+^ T-B cell conjugates, obtained from the spleen of a *wt* C57BL/6 control mouse. (b) Representative plots of naïve (top row) and infected (35 days post-infection, bottom row) of *Sh2d1a*^*−/−*^ and *wt* C57BL/6 control mice. Gates show frequency of CD3^+^ B220^+^ cells positive for CD19 and CD4. (c) Total numbers of CD3^+^ CD4^+^ B220^+^ CD19^+^ T-B cell conjugates in *Sh2d1a*^*−/−*^ and *wt* C57BL/6 control mice. (d) Representative plots of naïve (top row) and infected (35 days post-infection, bottom row) of *Sh2d1a*^*−/−*^ and *wt* C57BL/6 control mice. Gates show frequency of CD3^+^ CD4^+^ B220^+^ CD19^+^ T-B cell conjugates expressing low CD38 and high GL-7. (e) Total numbers of CD3^+^ CD4^+^ B220^+^ CD19^+^ CD38^lo^GL-7^+^ T-B cell conjugates in *Sh2d1a*^*−/−*^ and *wt* C57BL/6 control mice. (f) Representative plots of naïve (top row) and infected (35 days post-infection, bottom row) of *Bcl6*^*fl/fl*^*CD4-cre*^*+/*^^*−*^ and *wt* control mice. Gates show frequency of CD3^+^ B220^+^ cells positive for CD19 and CD4. (g) Total numbers of CD3^+^ CD4^+^ B220^+^ CD19^+^ T-B cell conjugates in *Bcl6*^*fl/fl*^*CD4-cre*^*+/*^^*−*^ and *wt* control mice. (h) Representative plots of naïve (top row) and infected (35 days post-infection, bottom row) of *Bcl6*^*fl/fl*^*CD4-cre*^*+/*^^*−*^ and *wt* control mice. Gates show frequency of CD3^+^ CD4^+^ B220^+^ CD19^+^ T-B cell conjugates expressing low CD38 and high GL-7. (i) Total numbers of CD3^+^ CD4^+^ B220^+^ CD19^+^ CD38^lo^GL-7^+^ T-B cell conjugates in *Sh2d1a*^*−/−*^ and *wt* C57BL/6 control mice. Bars represent median values. Data are representative or pooled from at least two independent experiments and were obtained in groups of 3–8 mice per time point. Statistical significance was obtained using Two-Way ANOVA with Tukey's multiple comparisons test. ND: Not done.

To study this, we used a method previously described (Reinhardt et al., 2009) to detect ex vivo stable B-T cell conjugates by flow cytometry using CD3, CD4, CD19 and B220 markers. CD3^+^ CD4^+^ CD19^+^ B220^+^ conjugates were approximately twice the size of single CD3^+^ CD4^+^ or CD19^+^ B220^+^ cells based on FSC detected by flow cytometry during *P. chabaudi* infection (Fig. 6a). The numbers of B cell-CD4^+^ T cell conjugates were not significantly altered in *Sh2d1a*^*−/−*^ mice compared with *wt* C57BL/6 controls either at day 15 or 35 p.i. (Fig. 6 b–c). However, the number of conjugates containing GC B cells (CD3^+^ CD4^+^ CD19^+^ B220^+^ CD38^+^ GL-7^+^), at day 35 p.i., were significantly lower in *Sh2d1a*^*−/−*^ mice compared with *wt* C57BL/6 controls at this time (Fig. 6 d–e). Albeit reduced, these interactions were still able to occur in SAP-deficient mice, as the number of GC B-CD4^+^ T cell conjugates in these mice was significantly higher at day 35 p.i. compared with background level (Fig. 6 d–e).

As expected, *Bcl6*^*fl/fl*^*CD4-cre*^*+/*^^*−*^ mice showed a significantly reduced number of B-T cell conjugates compared with *Bcl6*^*fl/fl*^ control mice at day 35 p.i. (Fig. 6 f–g), and no GC B-CD4^+^ T cell conjugates above background level at any time point studied (Fig. 6 h–i).

Thus, the lack of Tfh-cell responses render mice unable to sustain stable GC B-CD4^+^ T cell interactions, while the lack of SAP signaling impairs, but does not entirely abrogate, these important interactions.

### 3.6 The Lack of Tfh Cells and Deficiency of SAP Differentially Impact the Activation of *P. chabaudi*-specific IgG Responses

The GC response is a major source of antibody class switching. Therefore, we hypothesized that the differential impact in GC responses to *P. chabaudi* due to absence of SAP signaling or Tfh responses altogether might be translated in a differential impact on the IgG response to the parasite. This, in turn, could explain the differential capacity of *Bcl6*^*fl/fl*^*CD4-cre*^*+/−*^ and *Sh2d1a*^*−/−*^ mice to control the infection. In previous works, we have extensively characterized the kinetics and isotypes of *P. chabaudi*-specific antibody responses in C57BL/6 mice (Pérez-Mazliah et al., 2015; Achtman et al., 2007; Quin and Langhorne, 2001; Ndungu et al., 2009). The *P. chabaudi*-specific IgM response is not detected in circulation in C57BL/6 mice above background level before the first two weeks post-infection, whereas the *P. chabaudi*-specific IgG response in circulation, as well as MSP1-specific IgG antibody secreting cells in the spleen, are not detected above background level before the first three weeks post-infection. Therefore, we studied the *P. chabaudi*-specific IgM and IgG responses in *Bcl6*^*fl/fl*^*CD4-cre*^*+/−*^ and *Sh2d1a*^*−/−*^ mice from day 35 p.i. onwards.

**Fig. 7:**
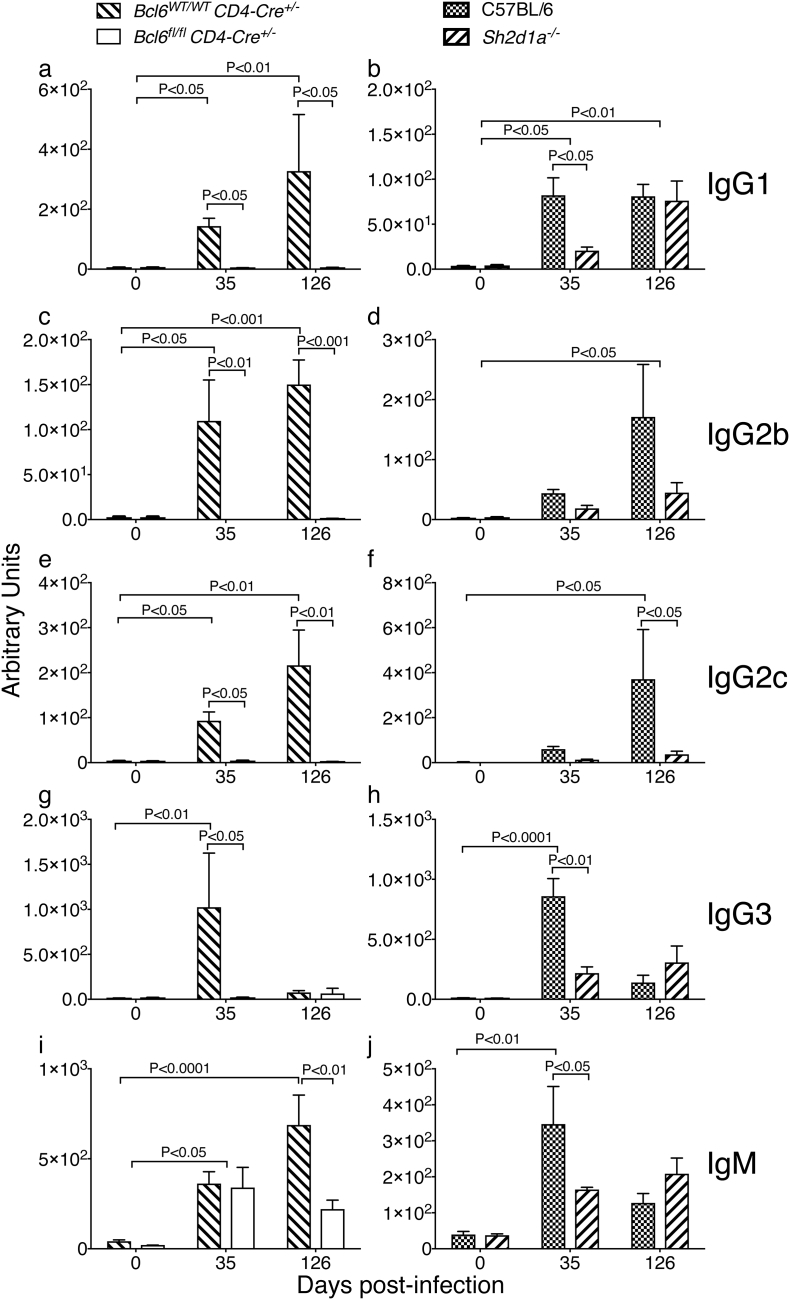
*Plasmodium chabaudi*-specific IgG responses are abrogated in *Bcl6*^*fl/fl*^*CD4-cre*^*+/−*^ mice, and partially altered in SAP-deficient mice. *Bcl6*^*fl/fl*^*CD4-cre*^*+/*^^*−*^ (left column) and *Sh2d1a*^*−/−*^ (right column) and respective *wt* control mice were infected with *P. chabaudi* via intra peritoneal injection of 10^5^ iRBC, and the levels of *P. chabaudi*-specific IgG1 (a–b), IgG2b (c–d), IgG2c (e–f), IgG3 (g–h) and IgM (i–j) were determined in serum samples by ELISA. Bars represent median values. Data are pooled from two independent experiments and were obtained in groups of 3–5 mice per time point. Statistical significance was obtained using Two-Way ANOVA with Tukey's multiple comparisons test.

In the absence of Tfh cells and GC B-cells, *Bcl6*^*fl/fl*^*CD4-cre*^*+/−*^ mice were unable to produce all subtypes of *P. chabaudi*-specific IgG antibodies (Fig. 7 a, c, e and g), whereas *P. chabaudi*-specific IgM responses were not altered up to day 35 p.i., and still detectable at day 126 p.i., in these mice (Fig. 7i). *P. chabaudi*-specific IgG responses were more variable in *Sh2d1a*^*−/−*^ mice; IgG2c antibodies were the most affected, while *P. chabaudi*-specific IgG1 and IgG3, as well as IgM, showed delayed kinetics in *Sh2d1a*^*−/−*^ mice compared with the antibody response of *wt* C57BL/6 control mice (Fig. 7 b, d, f, h and j). Therefore, both IgG and IgM responses were affected, but not completely abrogated by the lack of SAP signaling.

These data demonstrate that *P. chabaudi*-specific IgM responses can still be activated in the absence of Tfh responses, and that even partial alterations in *P. chabaudi*-specific IgG responses, as seen in SAP-deficient mice, are associated with reduced capacity to control this infection.

### 3.7 What Contributes to the Control of *P. chabaudi* Infection in the Absence of Tfh Cells?

Although the *Bcl6*^*fl/fl*^*CD4-cre*^*+/−*^ mice, completely deficient in Tfh cell and GC B-cell responses, failed to eradicate chronic *P. chabaudi* infection, these mice did not succumb to the infection, similar to mice with a complete deficiency of B cells do not succumb to *P. chabaudi* infection (von der Weid et al., 1996). These data strongly suggest that there is an additional immune component that, although not being able to eradicate the infection, is capable of controlling *P. chabaudi* to some extent.

**Fig. 8:**
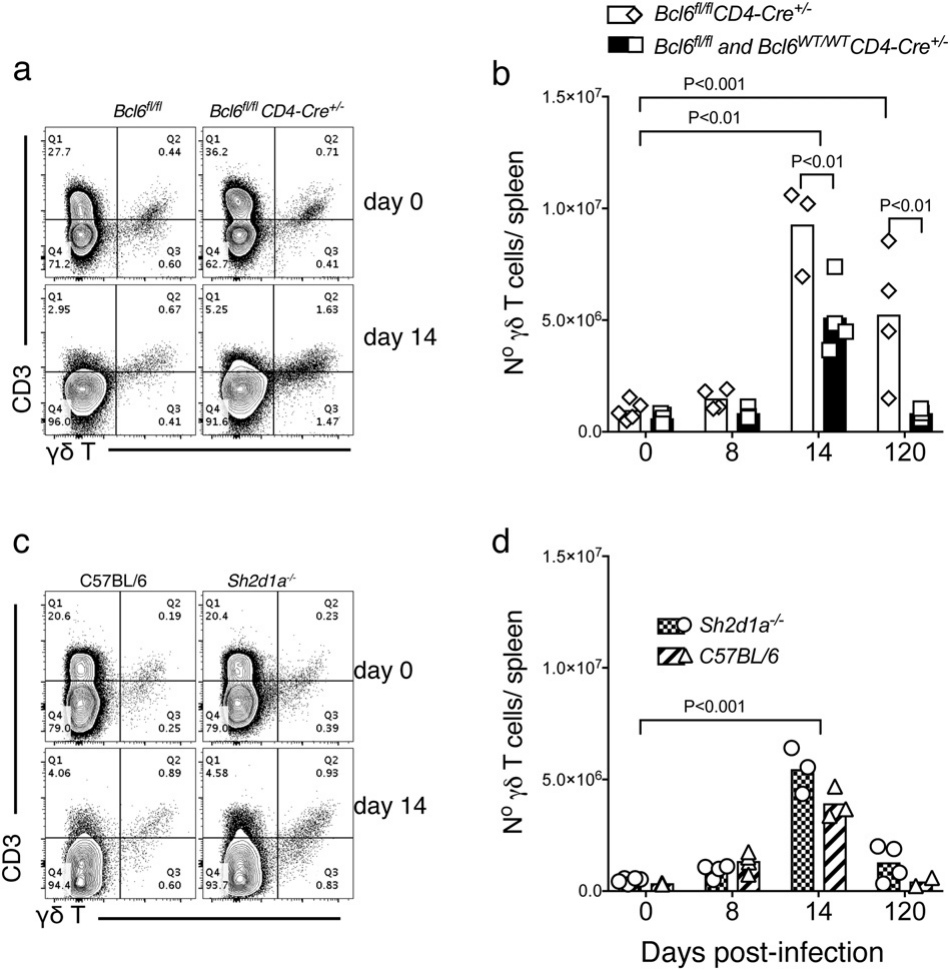
*Bcl6*^*fl/fl*^*CD4-cre*^*+/−*^ mice have increased numbers of γδT cells in the spleen during chronic *P. chabaudi* infection. *Bcl6*^*fl/fl*^*CD4-cre*^*+/*^^*−*^ and *Sh2d1a*^*−/−*^ mice were infected with *P. chabaudi* via intra peritoneal injection of 10^5^ iRBC, and the number of γδT cells in the spleen was determined by flow cytometry at days 0, 8, 14 and 120 post-infection. (a) Representative plots of naïve (top row) and infected (14 days post-infection, bottom row) *Bcl6*^*fl/fl*^*CD4-cre*^*+/−*^ and *wt* control mice. Upper right quadrants show frequency of CD3^+^ γδ^+^ T cells. (b) Total numbers of γδT cells in *Bcl6*^*fl/fl*^*CD4-cre*^*+/*^^*−*^ and *wt* control mice. (c) Representative plots of naïve and infected *Sh2d1a*^*−/−*^ and *wt* C57BL/6 control mice. (d) Total numbers of γδT cells in *Sh2d1a*^*−/−*^ and *wt* C57BL/6 control mice. Bars represent median values. Data are representative or pooled from at least two independent experiments and were obtained in groups of 3–5 mice per time point. Statistical significance was obtained using Two-Way ANOVA with Tukey's multiple comparisons test.

We have previously shown that μMT mice, deficient in B cells, undergo a dramatic expansion of the number of γδT cells in the spleen in response to *P. chabaudi* infection (von der Weid et al., 1996). Similarly, *Bcl6*^*fl/fl*^*CD4-cre*^*+/−*^ mice showed increased number of γδT cells in the spleen during the chronic phase of infection (Fig. 8 a–b). However, this was not the case for *Sh2d1a*^*−/−*^ mice (Fig. 8 c–d), probably due to the better control these mice have of the infection compared to *Bcl6*^*fl/fl*^*CD4-cre*^*+/−*^ mice.

**Fig. 9:**
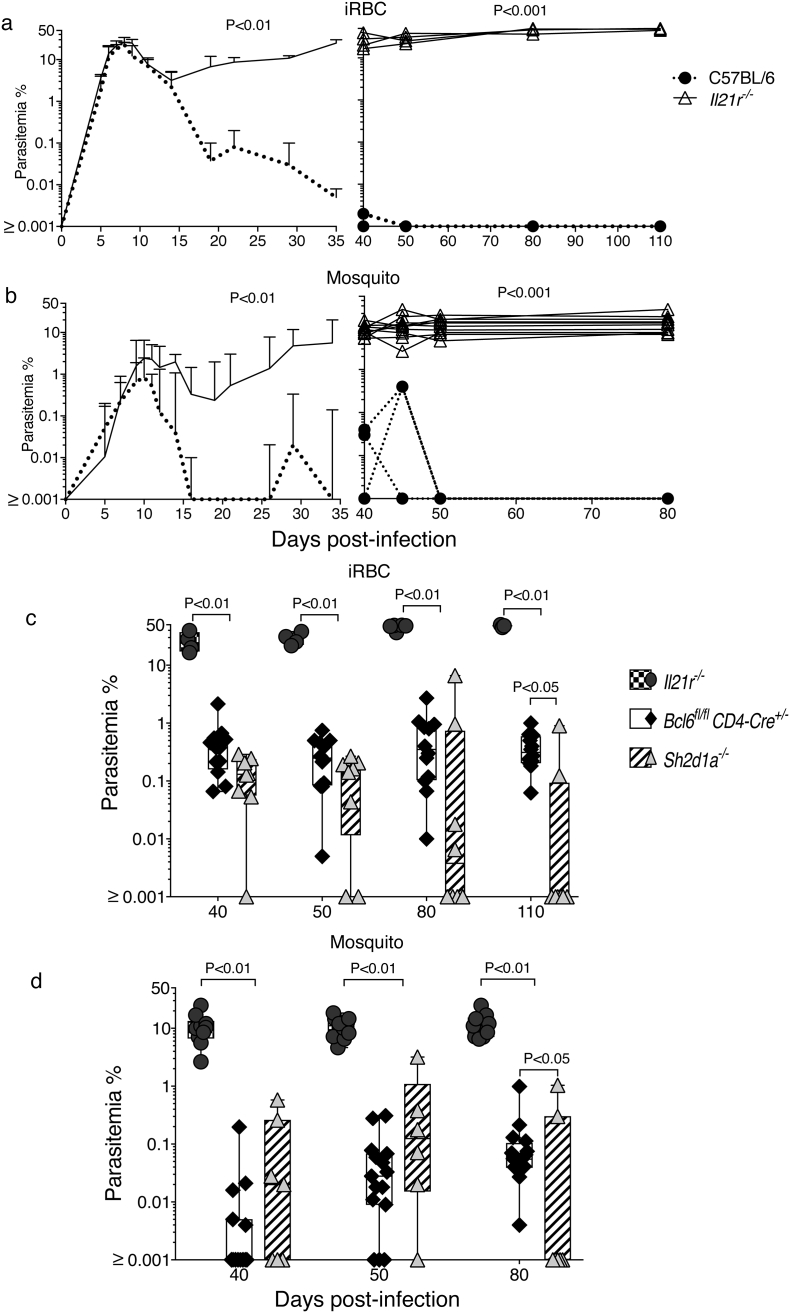
IL-21, Bcl6 and SAP are differentially required for the control of chronic *P. chabaudi* infection. *Il21r*^*−/−*^, *Bcl6*^*fl/fl*^*CD4-cre*^*+/−*^, *Sh2d1a*^*−/−*^ mice, and their respective *wt* control mice, were infected with *P. chabaudi* via intra peritoneal injection of 10^5^ iRBC or bites from *P. chabaudi*-infected mosquitoes and parasitemias were periodically monitored by giemsa-stained thin blood smears. The course of acute (left column) and chronic (right column) infection for *Il21r*^*−/−*^ mice following iRBC infection (a) and mosquito infection (b). The acute phase is displayed as median ± range, while curves corresponding to individual mice are displayed for the chronic phase. Cumulative data showing side-by-side the parasitemias during chronic *P. chabaudi* infection in *Il21r*^*−/−*^, *Bcl6*^*fl/fl*^*CD4-cre*^*+/*^^*−*^ and *Sh2d1a*^*−/−*^ mice, following iRBC infection (c) and mosquito infection (d). Data are representative of at least two independent experiments and were obtained in groups of 5–10 mice per time point. Statistical significance was obtained using Mann Whitney *U* test or Two-Way ANOVA with Tukey's multiple comparisons test.

**Fig. 10:**
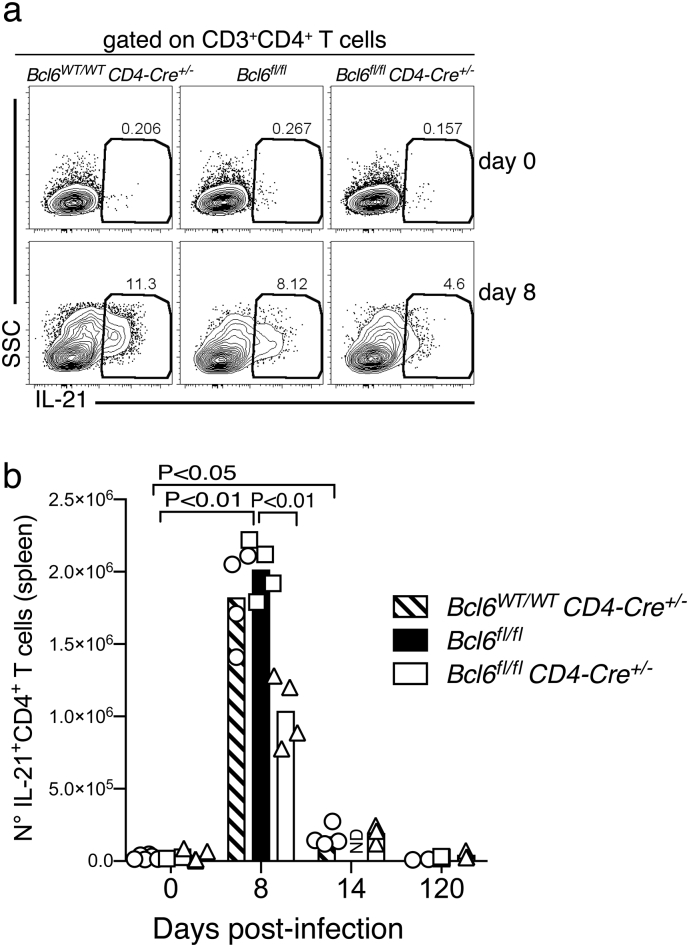
IL-21^+^ CD4^+^ T cells are activated in response to *P. chabaudi* infection even in complete absence of Tfh cells. *Bcl6*^*fl/fl*^*CD4-cre*^*+/−*^ mice were infected with *P. chabaudi* via intra peritoneal injection of 10^5^ iRBC, and the number of IL-21-producing CD4^+^ T cells in the spleen was determined by intracellular flow cytometry staining at days 0, 8, 14 and 120 post-infection. (a) Representative plots of naïve (top row) and infected (8 days post-infection, bottom row) *Bcl6*^*fl/fl*^*CD4-cre*^*+/−*^ and *wt* control mice. Gates show frequency of CD3^+^ CD4^+^ T cells expressing IL-21. (b) Total numbers of IL-21-producing CD4^+^ T cells in *Bcl6*^*fl/fl*^*CD4-cre*^*+/*^^*−*^ and *wt* control mice. Data are representative of two independent experiments and were obtained in groups of 3–5 mice per time point. Statistical significance was obtained using Two-Way ANOVA with Tukey's multiple comparisons test. ND: Not done.

We have recently shown that IL-21 is critical to activate protective B-cell responses to *Plasmodium* infection (Pérez-Mazliah et al., 2015). Similar to *Bcl6*^*fl/fl*^*CD4-cre*^*+/−*^ and *Sh2d1a*^*−/−*^ mice, *Il21r*^*−/−*^ mice had an initial course of acute *P. chabaudi* infection with peak parasitemias at days 8–9 (Fig. 9 a–b). Also, similar to *Bcl6*^*fl/fl*^*CD4-cre*^*+/−*^ and *Sh2d1a*^*−/−*^ mice and unlike *wt* C57BL/6 controls, *Il21r*^*−/−*^ mice failed to reduce parasitemia during the chronic phase, and the infection remained at levels between 10 and 50% from day 15 p.i. up to the 110 days of the experiment, without mortality [Fig. 9 a–b and (Pérez-Mazliah et al., 2015)]. These chronic parasitemias were notably higher than the chronic parasitemias observed in either *Bcl6*^*fl/fl*^*CD4-cre*^*+/−*^ and *Sh2d1a*^*−/−*^ mice (Fig. 9 c–d). Thus, intriguingly, it appears that IL-21 has a role in controlling *P. chabaudi* infection beyond Tfh-dependent B-cell activation. In accordance with this hypothesis, we have previously shown that the CD4^+^ T cell compartment producing IL-21 in response to *P. chabaudi* was approximately equally composed of Tfh and non-Tfh CD4^+^ T cells, and exclusively detected during acute infection (Pérez-Mazliah et al., 2015). In agreement, despite complete absence of Tfh cell activation, IL-21-producing CD4^+^ T cells were still activated in *P. chabaudi*-infected *Bcl6*^*fl/fl*^*CD4-cre*^*+/*^^*−*^ mice (Fig. 10). Moreover, as predicted, the number of IL-21-producing CD4^+^ T cells detected at the peak of infection in *Bcl6*^*fl/fl*^*CD4-cre*^*+/−*^ mice was reduced to approximately half the amount of these cells in *wt* controls, thus showing that the Tfh response represents an important, but not the only, source of IL-21 during *P. chabaudi* infection (Fig. 10).

In summary, we show that different signals previously implicated in B-T cell communication differentially impact the control of chronic *P. chabaudi* infection, following a gradient: IL-21 deficiency > Tfh deficiency > SAP deficiency.

## 4 Discussion

Our study formally demonstrates an essential requirement of Tfh cells to eradicate a chronic systemic *Plasmodium* infection initiated by natural vector transmission. In addition, we highlight substantial differences in the signaling network leading to GC activation in response to *Plasmodium* compared to immunizations or other infection models.

The effects of natural transmission by the mosquito in experimental models have generally been overlooked in studies of immune mechanisms elicited to blood-stage *Plasmodium* infection. However, immune responses, in particular B-cell responses, elicited to the less physiological injection of *Plasmodium*-infected red blood cells can differ from those elicited by natural transmission (Spence et al., 2013). Therefore, the natural vector route is an important component of the experimental system for host responses to *Plasmodium* parasites. Here, we validated all the responses observed using models of natural mosquito transmission.

Previous studies both in human and mouse models have shown the importance of B cell-responses to control *Plasmodium* infection (Cohen et al., 1961; Conway et al., 2000; Fowkes et al., 2010; Osier et al., 2008; Sabchareon et al., 1991; Burns et al., 1997; von der Weid et al., 1996). The signals required to activate protective B-cell responses to this infection have recently started to emerge. Tfh cells have been identified as the critical CD4^+^ T helper subset required for activation of follicular B-cell responses (Schaerli et al., 2000; Breitfeld et al., 2000; Kim et al., 2001). Recent evidence has suggested that *Plasmodium* infection has the capacity to bias the CD4^+^ T cell response toward a predominantly IFN-γ-producing Th1 response and consequently impair Tfh cell differentiation resulting in poor B-cell responses to the parasite (Hansen et al., 2017). Nonetheless, both Tfh cell and GC B-cell responses are strongly activated in a number of *Plasmodium* models (Zander et al., 2015; Matar et al., 2015; Achtman et al., 2003; Pérez-Mazliah et al., 2015; Wikenheiser et al., 2016), and in *P. chabaudi* infections these are not impaired by the activation of a strong Th1 response (Pérez-Mazliah et al., 2015; Wikenheiser et al., 2016). Herein, we investigated the requirement of Tfh cells to control *Plasmodium* infection in the *P. chabaudi* model. We focused on the transcription repressor Bcl6, the master regulator of the Tfh program (Johnston et al., 2009; Nurieva et al., 2009; X. Liu et al., 2009), and on the SAP molecule, previously shown to be required for B-Tfh cell interactions (Qi et al., 2008), and the activation of GC B-cells (Crotty et al., 2003; Cannons et al., 2006; Kamperschroer et al., 2006; Crotty et al., 2006; McCausland et al., 2007; Moyron-Quiroz et al., 2009; Yusuf et al., 2010; Morra et al., 2005) and Tfh cells in some models (Cannons et al., 2010; Deenick et al., 2010; Linterman et al., 2011). A targeted deletion of the *Bcl6* gene restricted to the T-cell lineage [*Bcl6*^*fl/fl*^*CD4-cre*^*+/−*^ (Kaji et al., 2012)] rendered mice unable to make Tfh cell responses to *P. chabaudi* infection and consequently unable to activate protective B-cell responses and eliminate the otherwise self-resolving blood stage of *P. chabaudi* infection. Of note, the Tfh cell response observed during the acute phase of *P. chabaudi* infection was critical to control the chronic, but not the acute, phase of *P. chabaudi* infection, as *Bcl6*^*fl/fl*^*CD4-cre*^*+/−*^ mice with a complete absence of Tfh cell responses were able to control the acute phase of infection without showing significant differences from the *wt* controls.

It is intriguing that there is a complete absence of all *P. chabaudi*-specific IgG subtypes in *Bcl6*^*fl/fl*^*CD4-cre*^*+/−*^ mice, including those associated with extra-follicular B cell responses. It is generally accepted that Bcl6 expression in T cells is required for extrafollicular IgG production (S. K. Lee et al., 2011). However, it is still a matter of debate as to how this happens, as Tfh cells by definition and functional capacity, preferentially home to the B-cell follicles (Qi, 2016). Nonetheless, a *P. chabaudi*-specific IgM response was readily detected even in complete absence of Tfh cell responses in *Bcl6*^*fl/fl*^*CD4-cre*^*+/−*^ mice. This demonstrates the activation of extra-follicular B cell responses to *P. chabaudi* in the absence of Tfh cells, and shows that an extra-follicular *P. chabaudi*-specific IgM response is not sufficient to eradicate *P. chabaudi* infection.

*Sh2d1a1*^*−/−*^ mice (lacking the gene that encodes SAP) were able to control the acute phase of *P. chabaudi* infection, but showed variable capacity to control the chronic phase of infection, and to completely eradicate the infection. Parasitemias determined by analysis of thin blood films at late time points post-infection (i.e. over 100 days post-infection) suggested clearance of parasites. However, more sensitive techniques to detect positive parasitemias [adoptive transfer of blood into *Rag2*^*−/−*^ mice (Achtman et al., 2007)] demonstrated the presence of sub-patent parasitemias in half of the *Sh2d1a1*^*−/−*^ mice studied.

Interestingly, the lack of SAP signaling did not alter the initial activation of Tfh cell responses to *P. chabaudi*, as demonstrated by the occurrence of normal levels of CD44^hi^CXCR5^+^ PD1^+^ as well as CXCR5^+^ Bcl6^+^ CD4^+^ T cells in the spleen of *P. chabaudi*-infected *Sh2d1a1*^*−/−*^ mice compared with C57BL/6 controls. The fact that the lack of SAP signaling did not disrupt the initial Tfh cell response to *P. chabaudi* seems to contradict some observations (Cannons et al., 2010; Deenick et al., 2010; Linterman et al., 2011). However, other works reported activation of Tfh cells in the absence of SAP, using both immunizations (Qi et al., 2008) and LCMV infection (Yusuf et al., 2010). Moreover, Deenick et al. demonstrated that the defect on Tfh activation in the absence of SAP occurs when the availability of antigen is limited, and can be rescued by boosting with antigen (Deenick et al., 2010). Therefore, the requirement of SAP for Tfh activation seems to be context-dependent, and the consensus in the field is now that SAP is not required for the initial activation of the Tfh program, but rather for the maintenance of this program and the relocalization of the Tfh cells into the germinal center (Crotty, 2014; Qi, 2012). This agrees with our observation of reduced, yet detectable, CD4^+^ GL7^+^ GC Tfh cells in the spleen of *P. chabaudi*-infected *Sh2d1a1*^*−/−*^ mice. Our observations of unaltered CD4^+^ T-B cell conjugates yet altered CD4^+^ T-GC B cell conjugates in *Sh2d1a1*^*−/−*^ mice compared with *wt* C57BL/6 controls, is in line with the long-lasting T-B cell interactions in the T-cell zone and follicle border and progressively shorter contact durations (and, therefore, more sensitive to mechanical disruption) inside the follicles and germinal centers observed in immunizations (T. Okada et al., 2005; Qi et al., 2008; Allen et al., 2007; Kerfoot et al., 2011; Shulman et al., 2014; D. Liu et al., 2015). On the other hand, SAP-deficient mice fail to activate GC B-cell responses to immunizations with T-dependent antigens, viral infections, and helminth infections (Crotty et al., 2003; Cannons et al., 2006; Kamperschroer et al., 2006; Crotty et al., 2006; McCausland et al., 2007; Moyron-Quiroz et al., 2009; Yusuf et al., 2010; Morra et al., 2005). Therefore, it is indeed surprising that SAP-deficient mice can activate, albeit significantly reduced, a distinctive GC response to *P. chabaudi*. This is demonstrated by the occurrence of a) CD38^low^GL7^+^ GC B cells, b) GC structures as determined by histology c) GL7^+^ CG Tfh cells, d) T cells within the GC as determined by histology, e) stable T-GC B cell conjugates and f) *P. chabaudi*-specific IgG responses in *Sh2d1a1*^*−/−*^ mice. The deficient T-B cell interaction driven by the absence of SAP can be partially compensated by other signals, i.e. strong cognate interactions in the context of high antigen availability on B cells (Qi, 2012; Qi et al., 2008). Thus, these mechanisms might be in place during *P. chabaudi* infection and able to partially compensate for the lack of SAP signaling. *P. chabaudi* infection is the only infection model identified to date in which GC B cells can be activated in the absence of SAP. A recent report by Wikenheiser et al. shows that ICOS is dispensable during early Tfh cell differentiation in response to *P. chabaudi* infection, but it is required for sustaining the Tfh cell response over time (Wikenheiser et al., 2016). Interestingly, both *P. chabaudi*-specific B-cell responses and the course of *P. chabaudi* infection in ICOS-deficient mice resemble our observations in SAP-deficient mice more closely than in *Bcl6*^*fl/fl*^*CD4-cre*^*+/−*^ mice (i.e. partial, but not complete, abrogation of the GC B cell response and *P. chabaudi*-specific IgG responses, and highly variable control of the parasitemia late during chronic infection) (Wikenheiser et al., 2016). ICOS engagement promotes persistent T-cell migration at the border between the T-cell zone and the B-cell follicle, and controls follicular recruitment of activated T-helper cells independently from the ICOSL-mediated co-stimulation provided by cognate B cells, thus acting as a molecular linkage between T–B cells (Xu et al., 2013; D. Liu et al., 2015; Qi, 2016). Therefore, it is possible that SAP and ICOS act via a similar mechanism during *P. chabaudi* infection, i.e. via regulating T helper cell positioning in the follicle and favoring T-B cell interactions, and can partially compensate for each other. Notably, the deficiency of ICOS signaling reduced GC B-T cell interactions during *P. chabaudi* infection, similarly to SAP deficiency (Wikenheiser et al., 2016). This inefficient, yet distinctive, GC activation in *Sh2d1a1*^*−/−*^ mice can explain the better control of the infection by these mice compared with the complete absence of Tfh cell responses in *Bcl6*^*fl/fl*^*CD4-cre*^*+/−*^ mice, and strongly supports a model in which the quality of the T-dependent B cell response reflects the capacity to control the blood stages of *P. chabaudi* infection.

In conclusion, here we provide evidence of the major role of Tfh cell responses in controlling and clearing the chronic phase of *Plasmodium* infection. In addition, we delineate a hierarchical requirement of signals previously associated with T-B cell interactions in the control of this infection, following the order IL-21 > Tfh cells > SAP. This proved to be true for both, blood and natural mosquito transmission of *P. chabaudi* infection. Intriguingly, these data suggest a role for IL-21 beyond B cell activation driven by Tfh cells. In agreement with this, c-Maf, but not Bcl6, is known to control IL-21 expression (Bauquet et al., 2009; Hiramatsu et al., 2010; Kroenke et al., 2012), and non-Tfh IL-21-producing CD4^+^ T cells are detected during *P. chabaudi* infection (Pérez-Mazliah et al., 2015; Carpio et al., 2015) and *P. berghei* ANKA infection (Ryg-Cornejo et al., 2015). Therefore, further research is needed to determine the full role of this pleiotropic cytokine in the control of this infection. Monitoring of both IL-21 and Tfh responses have generally not been included in studies aimed at generating protective vaccines to *Plasmodium* infection. In the light of data presented herein, monitoring of both of these critical immune signals should be considered when evaluating the efficacy of novel immune therapies designed to prevent or eliminate *Plasmodium* infection.

Our data complete the study of a battery of fundamental signals implicated in T-B cell interactions in the context of blood-stage *Plasmodium* infection, i.e. PD-1 (Butler et al., 2012; Zander et al., 2015), IL-21 (Pérez-Mazliah et al., 2015), OX40 (Zander et al., 2015) and ICOS (Wikenheiser et al., 2016). Altogether, these data provide a framework to weigh the relevance of different signals regulating T-B cell interactions for the control of this complex parasite, which is critical to reach the goal of generating protective immunotherapies to control the spread of malaria.

The following are the supplementary data related to this article.

## Funding Sources

This work was supported by the Francis Crick Institute which receives its core funding from the UK Medical Research Council (FC001101), Cancer Research UK (FC001101) and the Wellcome Trust (FC001101); by the Wellcome Trust (grant reference WT101777MA). Irene Tumwine is the recipient of a Francis Crick PhD studentship. The funding bodies played no role in the design or interpretation of the experiments, or the decision to submit for publication.

## Conflicts of Interest

All authors, no conflicts of interest.

## Author Contributions

Conceived and designed the experiments: DPM JL. Performed the experiments: DPM MPN CH SM IT PH. Analyzed the data: DPM MPN CH SM ML JL. Contributed reagents/materials/analysis tools: JL. Wrote the paper: DPM JL.
